# The Moderating Role of Sociability and Social Connection for the Relationship between Soccer Participation and Teamwork Ability among Chinese College Students

**DOI:** 10.3390/ijerph192315441

**Published:** 2022-11-22

**Authors:** Yuetao Liu, Songhui You, Zhiyuan Wang

**Affiliations:** 1 College of Humanities Education, Yancheng Kindergarten Teachers College, Yancheng 224005, China; 2 International College of Football, Tongji University, Shanghai 200092, China

**Keywords:** soccer participation, teamwork ability, sociability, social connection, moderating effect

## Abstract

In order to provide empirical evidence for soccer’s promotion of teamwork ability and to examine whether sociability and social connection have an effect on this promotion, we explored the relationship between soccer participation (volume, duration), teamwork ability and sociability and social connection (SSC). Using the method of stratified sampling, a questionnaire survey was carried out in four universities in Shanghai. All the respondents are undergraduate students, which include the specially recruited soccer athletes and the soccer participants from ordinary college students. The findings from this study indicate that participating in soccer can positively predict the teamwork ability of college students, and SSC can negatively moderate the effect of soccer participation on teamwork ability. The effect of soccer participation on teamwork ability was different in the collegiate soccer athletes and collegiate soccer participants groups. An important value of soccer, which is often overlooked, is the help it provides college students, who have insufficient sociability and social connections, in better integrating into the team and in improving their teamwork ability. We highly recommend that college students participate in soccer to improve their teamwork skills in study and work and to better prepare for their careers.

## 1. Introduction

One of the main goals of a college education is to develop the practical skills necessary for the career development of college students, helping them prepare for a career path after graduation [1]. Nowadays, simple repetitive tasks and manual labor are gradually replaced by automatic computer programs and algorithms [2], and by and large, people need to solve increasingly complicated problems through innovation and cooperation [3]. In this context, teamwork has always been recognized as a key competency for success in various fields [4,5]. The development of teamwork skills is an essential direction for higher education research [6,7]. Many colleges have identified teamwork as a basic employment skill and have regarded it as an important part of student assessment. Given its importance for practitioners in many arenas, teamwork ability has become an increasingly popular topic that fascinates researchers from diverse social science fields [8].

As a collective ball game, soccer has always been closely linked with teamwork [9,10,11]. It has become a general consensus that putting together a group of highly skilled players on a team does not mean championships [8]. In soccer, while some member tasks are independent behaviors and others are interdependent, it is essential to work in synergy with teammates in order for a team to work optimally. Teamwork is a fundamental principle for teams to achieve their best performance [12]. On a soccer team, for instance, a player who can only dribble and shoot on his own is far from a truly valuable player; he also needs to be able to collaborate with his teammates so that the team can benefit from the unique abilities of each member [13]. In addition to its instrumental value, teamwork also represents a certain athletic virtue. One of the first lessons in soccer is the importance of teamwork, i.e., a “good team player” seems to connote something nobler than “a talented player” or even “a well-trained player” [14]. The values of perfectionism [15], collectivism and equality [14] provide an ethical context for teamwork in soccer and explain why soccer is particularly suitable for youth education. Studies of Spanish soccer players support the fact that the motivational climates, task climates, created by coaches and cohort members are associated with team cohesion, satisfaction [16].

Teamwork has attracted so much attention in the field of soccer and other sports that it has been increasingly valued in work and study as well. Teamwork in soccer serves as a model for some “real life” lessons, a model that is extended to general issues of human society [14]. People seem to have long been accustomed to taking soccer’s shaping and promotion of teamwork ability for granted. However, when we tried to find the associated empirical evidence, we found that the majority of research was on how to improve the teamwork level of a soccer team; thus, there was a lack of research on the effect of soccer participation on people’s teamwork ability. Comparisons between the specially recruited soccer athletes and the soccer participants from ordinary college students are especially and notably missing. Thus, the contribution of soccer participation to enhancing teamwork ability needs to be critically examined with empirical data and presented visually.

Teamwork is built on people-to-people communication [17]. Stevens and Campion [18] divide teamwork ability into two dimensions: interpersonal KSA (knowledge, skills and abilities) and self-management KSA. Interpersonal KSA includes conflict resolution, cooperative problem resolution and communication [18]. Sociability and social connection are closely related to teamwork ability [19,20]. The concept of social connection describes individuals’ personal relationships and networks or the connections people have with other groups [21]. Social connection could encompass not only the connections with families and friends but also the ones made at work or through different activities [22]. Sociability is defined as people’s capacity and tendency to be sociable, to recognize and respond positively to others’ mental states [23]. Sociability is a key factor that affects social connection. The interaction between the two is inseparable, making them suitable to be combined as one variable for research [21]. In a team, we must not only have the ability and willingness to accomplish team tasks (task attributes of a team) but must also focus on team members’ harmonious interaction and collaboration, which relies on the team members’ sociability and social connection [24]. Good sociability and social connection are the personal foundation of good teamwork [25,26,27], which has a good role in promoting individual performance and internal relationship-building on the team. The realization of social attributes will not only enable individuals of the team to achieve a better job status but will also agglomerate the identification and input of the whole team to the job, leading to an efficient team [28].

There are also studies that provide evidence for the correlation between soccer participation, sociability and social connection [29]. Soccer is not only a sport but also a culture, an education and, more, a social life atmosphere. Prolonged participation in soccer can benefit those who are socially estranged [30]. A Korean study of infants showed that physical activity among peers can help infants establish good relationships and positively affect infants’ social development [31]. Some Chinese scholars have studied soccer as an exercise prescription to address interpersonal distress among college students. The researchers argue that soccer provides college students with places and opportunities for interaction needs, enabling them to: overcome loneliness in United and collaborative multidirectional communication; generate feelings of closeness and trust with others; expand social contacts; and thus, improve college students’ social resilience and avoid psychological discomfort caused by poor interpersonal relationships [32,33]. Communication and mutual support in soccer, as well as the facilitation of emotional control and the amelioration of mood, are suggestive of a role for soccer participation in contributing to increased sociability that is well worth exploring. The current research on the relationship between the three is still missing, and thus, further exploration is required.

Building on previous research, we aim to provide empirical evidence for soccer’s promotion of teamwork ability. On the basis of validating the relationship between soccer participation and teamwork ability, we further explore the role of sociability and social connection. The differences and connections between the specially recruited soccer athletes and the soccer participants from ordinary college students are also explored. To this end, we take college students as subjects and propose the following hypotheses:

**H1:**  *Volume can significantly and positively predict teamwork ability.*

**H2:**  *Duration can significantly and positively predict teamwork ability.*

**H3:**  *Volume can significantly and positively predict SSC.*

**H4:**  *Duration can significantly and positively predict SSC.*

**H5:**  *SSC can significantly and positively predict teamwork ability.*

**H6:**  *SSC can play a moderating role between volume and teamwork ability.*

**H7:**  *SSC can play a moderating role between duration and teamwork ability.*

**Figure 1:**
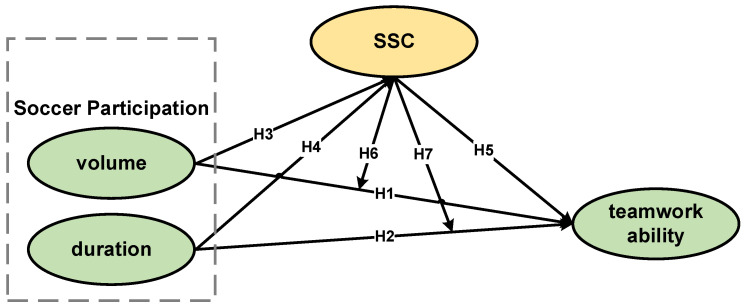
Hypothetical model.

The hypothetical model is shown in Figure 1.

## 2. Materials and Methods

### 2.1. Measures

#### 2.1.1. Soccer Participation

This study measures the dimension of soccer participation from the perspectives of the usual exercise volume of soccer (volume) and the exposure duration to soccer (duration). Volume was measured using The Physical Activity Scale–3 (PARS–3) [34,35]. A Likert scale of one to five is used for scoring. PARS–3 includes three dimensions: frequency of weekly exercise; time of each exercise; and intensity of each exercise. Volume is the product of the scores in the three dimensions. This scale is used to measure the level of soccer participation at the current stage or at a specific stage in the past. The test–retest reliability of the PARS–3 is 0.82, and the Cronbach α is 0.83. In order to include soccer career or historical soccer experience in the measurement, duration is measured by the exposure duration to soccer. Due to the fact that the level of volume is not constant at different stages of college students’ soccer career, it is impossible to continue to conduct cumulative calculation together with frequency, time and intensity. Therefore, volume and duration are considered as two dimensions.

#### 2.1.2. Teamwork Ability

Teamwork ability is measured using Team–Q [1], which is simplified and compiled based on Team Up. Team–Q is organized into five categories, totaling fourteen items. Measured using a Likert scale from 1 (never) to 5 (always). The test–retest reliability of the Team–Q is 0.80, and the Cronbach α is 0.96.

#### 2.1.3. Sociability and Social Connection

The Personal Sociability and Connections Scale (PeSCS) [21] are used to measure sociability and social connection (SSC). The scale consists of 10 short questions with responses on a Likert scale from 1 (never) to 4 (always), which are divided into three sections: social, behavioral and emotional. We adjusted the scale range from 1–4 to 1–5 to facilitate data analysis. The test–retest reliability of the PeSCS is 0.93, and the Cronbach α is 0.88.

### 2.2. Participants and Procedure

Using the method of stratified sampling, a questionnaire survey was carried out in four universities in Shanghai (Tongji University, University of Shanghai for Science and Technology, Donghua University and Shanghai University). All the respondents are undergraduate students, which include the specially recruited high-level soccer athletes (hereinafter referred to as collegiate soccer athletes) and the soccer participants from ordinary college students (hereinafter referred to as collegiate soccer participants). Since the number of collegiate soccer participants is much larger than that of collegiate soccer athletes and the sampling proportions have no effect on the results of this study, there is no need to control for equal sampling proportions. The distribution methods of the questionnaire include in-team distribution by team coaches, random distribution on the soccer field and random distribution online. In preparation for the questionnaire survey, we conducted a test–retest reliability test with 60 samples. After the formal survey began, we first sent invitation letters to all respondents. Besides the introduction of the research and the obligations of the investigator and respondent, the invitation letter also contained the Informed Consent Form. Responses to the questionnaire survey were permitted only after the respondent had read and accepted the information contained in the Informed Consent Form. All respondents filled in the questionnaire online through Wenjuan.com. Online questionnaires can effectively avoid missing questions and record information such as the duration of answers. The study was approved by the Tongji University Ethics Committee and complied with all ethical regulations. This study covered filling questionnaires only; no biological intervention on humans was conducted. All the interviewers expressed their will to take part in the study and signed informed consent forms.

A total of 35 items are included in the questionnaire, including three scales and demographic information. As part of the questionnaire, two screening items are included to identify invalid responses. Simultaneously, invalid questionnaires were eliminated if the answering time is less than one minute. We received 629 questionnaires, of which 543 were valid, resulting in an 86.33% effective rate. The number of people in the collegiate soccer athletes group was 67, and the number in the collegiate soccer participants group was 476. According to the valid questionnaires, 31.5% (n = 171) of the sample were females, and 68.5% (n = 372) were males. The average age of participants was 19.79 years (SD = 1.52, range 16–27).

A principal component analysis is carried out using Harman’s single-factor test method [36]. It is found that there are five factors with characteristic roots above one, and the first factor explains 28.58% of the variation, which is less than the critical level of 30%. Due to this, the common method bias needs not be taken into consideration.

To test the correlation degree of the questionnaire data, KMO and the Bartlett Test of Sphericity were performed. The KMO value is higher than 0.8, being 0.934. Bartlett’s test has a *p*-value of 0.000, which meets significance criteria. As a result, the data in the scales are very suitable for factor analysis. To test whether the questionnaire was consistent with our design, a factor analysis was performed using SPSS. Five factors with initial eigenvalues over one are extracted through principal components analysis. The five factors explained 73.39% of the cumulative variance variation. Therefore, five factors, including volume, SSC, teamwork ability and their different dimensions, are eventually extracted. The factor loadings of the five factors are all above 0.6. Factors from the same variable were combined and tested for validity. The obtained AVE value was above 0.5; the CR value was above 0.7. The test results of the questionnaire met the validity requirements.

In order to test the correlation of each variable separately, the Pearson correlation analysis was performed using SPSS. The mean and standard deviation of the variables were calculated. Regression analysis was performed using SPSS. The regression models are plotted with the help of GraphPad. Moderating effects tests were performed using the PROCESS macro based on ordinary least-squares regression [37]. The Johnson–Neyman (J–N) technique was used to find and visualize regions with significant moderating effects [38]. ANOVA was used to test the differences between collegiate soccer athletes and collegiate soccer participants and to verify whether there was a significant moderating effect in both groups, and the moderating effect test was performed in the two groups.

## 3. Results

### 3.1. Descriptive Statistics and Correlations

Table 1 shows the results of the Pearson analysis. The results show that there is a significant correlation between volume and teamwork ability, and there is no correlation between volume and SSC; the hypothesis H3 does not hold. Duration also had a significant correlation with teamwork ability but not with SSC; the hypothesis H4 does not hold. In addition, a significant correlation exists between SSC and teamwork ability. Using SPSS to perform regression analysis on SSC and teamwork ability, the results support that SSC can significantly, positively predict teamwork ability; the hypothesis H5 is verified. This shows that SSC cannot play a mediating role.

### 3.2. Moderating Effect of SSC

To test whether volume and duration have a significant effect on teamwork ability, the regression analysis was carried out with gender as the control variable and SSC as the moderator variable. As shown in Table 2, except for the control variable, the other significance levels are less than 0.05, indicating that the regression model has passed the significance test. Since R^2^ = 0.257, the model’s interpretation of the original data is ideal. The results show that volume can significantly, positively predict teamwork ability; the hypothesis H1 is verified. The regression models for duration and teamwork ability were tested in the same way, yielding the identical results (F = 45.164, R^2^ = 0.251); the hypothesis H2 is verified.

**Figure 2:**
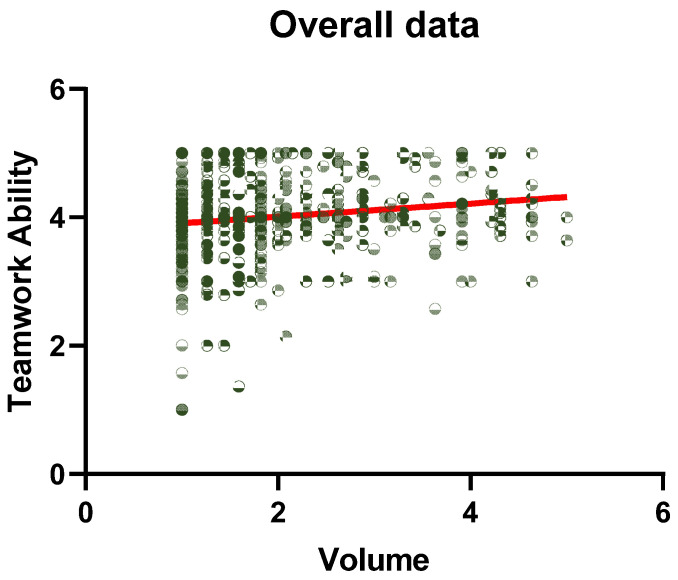
Regression model. Each dot represents a sample, and the darker the color, the greater the number of samples at that dot. The line reflects the trend of the dependent variable with the independent variable.

As shown in Figure 2, the regression model of volume and teamwork ability was drawn using GraphPad.

The significance of the moderation model was tested using Model 1 of the PROCESS macro proposed by Hayes [37,39]. The results showed that the direct effect of volume on teamwork ability was significant (β = 0.078, *p* < 0.01, 95% CI [0.026, 0.130]). The moderating effect of SSC was also significant (β = −0.105, *p* < 0.05, 95% CI [−0.191, −0.020]). The hypothesis H6 is verified. This means that SSC can play the role of a negative moderator between volume and teamwork ability. The regression equation is expressed as follows:

teamwork ability = 0.078 × volume + 0.549 × SSC − 0.105 × volume × SSC + 4.004(1)

**Figure 3:**
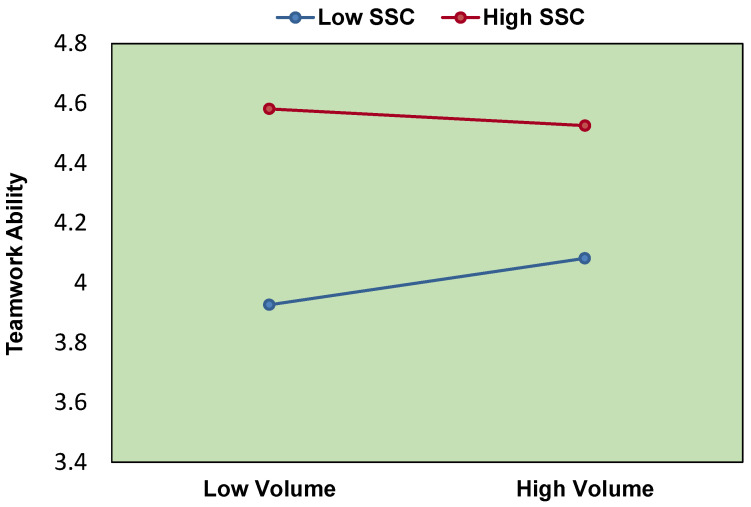
The moderating effect of SSC.

To illustrate the moderating effects of SSC, we plotted the regression of volume on teamwork ability at high and low levels of SSC (high, above the median; low, below the median) (see Figure 3). The results show that SSC can play the role of a negative moderator between volume and teamwork ability. At low SSC level, volume can significantly and positively predict teamwork ability, but at high SSC level, the predictive effect of volume on teamwork ability is no longer significant.

**Figure 4:**
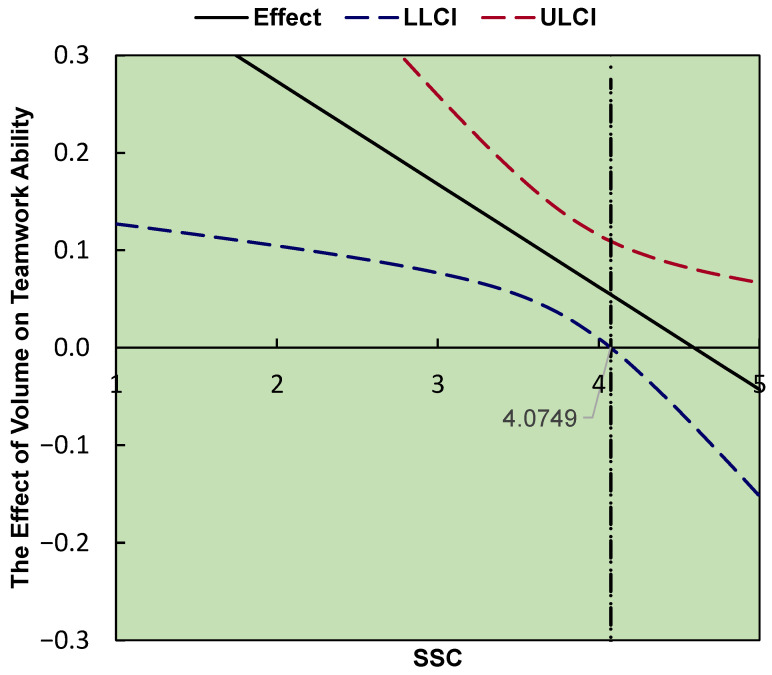
J–N. The dotted line “ULCI” represents the upper limit of the confidence interval, and “LLCI” represents the lower limit of the confidence interval. The line “Effect” represents the effect of volume on teamwork ability.

As shown in Figure 4, the J-N technique indicated that the score of 4.075 on SSC can be regarded as a point of transition between significant and nonsignificant. When the SSC score is lower than 4.075, volume can significantly and positively predict teamwork ability.

The same moderation effect testing procedure was performed with duration as the independent variable. The results showed that the direct effect of duration on teamwork ability was significant (β = 0.043, *p* < 0.05, 95% CI [0.009, 0.078]), and the moderating effect of SSC was also significant (β = −0.059, *p* < 0.05, 95% CI [−0.117, −0.001]). The hypothesis H7 is verified. This means that SSC can play the role of a negative moderator between duration and teamwork ability. The following is the regression equation expression:

teamwork ability = 0.043 × duration + 0.552 × SSC − 0.059 × duration × SSC + 4.002(2)

### 3.3. Comparison of Collegiate Soccer Athletes and Collegiate Soccer Participants

First, the differences between the two groups were tested by ANOVA. The results showed that collegiate soccer athletes were significantly higher in volume and duration than collegiate soccer participants, but there were no significant differences in SSC and teamwork ability. Then, collegiate soccer athletes and collegiate soccer participants were tested for moderation effect. In the collegiate soccer athletes group, the direct effect of volume on teamwork ability was not significant (β = 0.055, *p* = 0.588, 95% CI [−0.146, 0.256]), and the moderating effect of SSC was not significant (β = −0.139, *p* = 0.380, 95% CI [−0.452, 0.175]). In the collegiate soccer participants group, the direct effect of volume on teamwork ability was significant (β = 0.113, *p* < 0.01, 95% CI [0.035, 0.190]), and the moderating effect of SSC was also significant (β =−0.145, *p* < 0.05, 95% CI [−0.265, −0.025]).

The identical procedure was performed with duration as the independent variable. In the collegiate soccer athletes group, the test result of duration as an independent variable is completely consistent with that of volume as an independent variable. But in the collegiate soccer participants group, the situation was different. The direct effect of duration on teamwork ability was not significant (β = 0.043, *p* = 0.063, 95% CI [−0.002, 0.088]), and the moderating effect of SSC was also not significant (β = −0.060, *p* = 0.095, 95% CI [−0.131, 0.011]).

## 4. Discussion

The results of this study indicate that participating in soccer can positively predict the teamwork ability of college students, which proved H1 and H2. This is consistent with the conclusions of the previous study of Spanish soccer players [16]. This finding provides empirical evidence for the view that soccer participation improves teamwork ability. Moreover, the effect of soccer participation on teamwork ability was different between the collegiate soccer athletes and collegiate soccer participants groups. On the one hand, playing soccer can no longer significantly improve their teamwork ability in study and work for the athletes in college, and on the other hand, participation in soccer is closely related to their teamwork ability for ordinary people and enthusiasts. The subjects’ soccer participation in this study was measured from two aspects: the usual exercise volume of soccer and the exposure duration to soccer. Based on the validated H1, a positive correlation exists between collegiate soccer participants’ usual exercise volume of soccer and their teamwork ability. When analyzing the relationship between duration and teamwork ability on the basis of the whole sample, the results show that duration is positively correlated with teamwork ability. However, when the relationship between duration and teamwork ability was analyzed separately based on the two sample groups of collegiate soccer athletes and collegiate soccer participants, the results showed that the effect of duration on teamwork ability was not significant in either group. We anticipate the following reasons. For Chinese specially recruited, high-level soccer athletes, all of them have received no less than 5 years of professional soccer training and even have been in contact with soccer for generally more than 10 years [40,41]. There are little samples with a short exposure duration. After a long period of professional training, the marginal effect of duration may be decreasing. For the soccer participants from ordinary college students, duration may not accurately reflect the amount of soccer experience they have. Based on the return visit, we found in our research that respondents who have been in contact with soccer for more than 10 years only played soccer when they were children and did not continue to play soccer afterwards. Compared to the specially recruited high-level soccer athletes, the diversity of the soccer participants from college students and the difference of self-measurement standard of soccer participation made the collegiate soccer participants’ duration less accurate. Measuring soccer participation from both volume and duration provided theoretical experience for future relevant research.

The results of this study showed no significant correlation between soccer participation and SSC; H3 and H4 were not validated. Compared with previous research [30], which showed the association between soccer participation and SSC is not strong enough, the authors guess that the difference in measurement dimensions (volume and duration were used in this study) and the difference in sampling (different participating populations) will lead to the different results. More accurate and comprehensive soccer involved measurement tools need to be developed in the future, with separate studies for different populations to clarify the relationship between soccer participation and SSC.

There is a significant correlation that exists between SSC and teamwork ability; H5 is verified. This proves that SSC is closely related to teamwork ability; the conclusion is consistent with the results of previous related studies [18,19,20,21,22]. This provides evidence for college students’ SSC and teamwork ability relationships under soccer scenarios, enriching the breadth of the theory.

SSC can negatively moderate the effect of soccer participation on teamwork ability; H6 and H7 were validated. This is an important finding of this study, which fills a gap in this research area and reveals the moderating role of SSC between soccer participation and teamwork ability; it also sheds light on the relationship between the three. As the SSC becomes higher, the predictive effect of soccer participation on teamwork ability decreases. When the SSC score exceeds 4.07, soccer participation’s predictive effect on teamwork ability is no longer significant. This shows that when a person’s SSC score is high, volume and duration have little effect on his teamwork ability. Because there is a high correlation between SSC and teamwork ability, people with high SSC scores generally have high teamwork ability scores, and soccer participation does not have much effect on their teamwork ability. But when an individual’s SSC score is low or middle, soccer participation can significantly improve teamwork ability. Therefore, an important value of soccer is to help college students with insufficient sociability and social connections to better integrate into the team and improve their teamwork ability.

Although not one of the hypotheses nor the main conclusion, one finding that has to be mentioned is: There was no difference in teamwork ability between collegiate soccer athletes and collegiate soccer participants. Based on the previous analysis, the more soccer involved, the better the teamwork ability they had. But athletes’ higher levels of soccer participation did not significantly advance their teamwork skills score over other ordinary college students. This may be because the educational experiences and growth paths of Chinese specially recruited high-level athletes and ordinary college students are completely different [42], and it is difficult to directly compare them to draw relevant conclusions.

## 5. Conclusions

The present study obtained a series of conclusions, first confirming that participating in soccer can improve the teamwork ability of college students. Second, high scores in SSC are associated with good teamwork ability among college students. Third, the most important finding of this study is that SSC can negatively moderate the strength of the main effect; as SSC increased, soccer’s effect on teamwork ability gradually decreased. Lastly, the effect of soccer participation on teamwork ability was different between the collegiate soccer athletes and collegiate soccer participants.

At the same time, we should also acknowledge the deficiencies of this study: On the one hand, this paper uses cross-sectional data. Although logically speaking, teamwork ability does not make a decisive difference to the degree of soccer participation; causality between the variants was not verified in a statistical sense. On the other hand, this paper has mainly adopted subjective reporting data for research, which remains to be explored through experimental data to reveal the effects of soccer participation on teamwork ability. For future studies, the authors recommend obtaining data experimentally so that causal mechanisms can be better confirmed. Besides, more accurate and comprehensive soccer involved measurement tools need to be developed in the future.

This paper reveals the moderating role of sociability and social connection between soccer participation and teamwork ability for the first time. This study provides a theoretical supporting and opens up a new idea for the research path, such as soccer participation can improve the fundamental ability of college students. For those with a low level of SSC, soccer participation has a particularly pronounced effect on improving teamwork ability. We highly recommend that college students participate in soccer to improve their teamwork skills in study and work and to better prepare for their careers. The colleges and universities are also encouraged to drive more college students to participate in soccer through physical education classes and club activities.

## Figures and Tables

**Table 1:** Descriptive Statistics and Correlations.

Variable	M	SD	Correlations			
			1	2	3	4
1. volume	1.893	1.007	1			
2. duration	2.120	1.503	0.811 **	1		
3. SSC	3.857	0.612	0.079	0.060	1	
4. teamwork ability	3.999	0.712	0.145 **	0.120 **	0.484 **	1

Note. ** *p* < 0.01.

**Table 2:** Moderated regression analysis.

Variable	Teamwork Ability		Teamwork Ability	
	β	t	β	t
Gender	0.008	0.184	0.057	1.520
volume			0.717	2.961 **
SSC			0.654	8.298 ***
volume × SSC			−0.649	−2.527 *
F	0.034		46.599	
R^2^	0.000		0.257	

Note. *** *p* < 0.001, ** *p* < 0.01, * *p* < 0.05.

## Data Availability

The data presented in this study are available on request from the corresponding author. The data are not publicly available due to confidentiality.
